# Hospital to Home Transitions for Children With Medical Complexity: A Scoping Review of Healthcare Professionals Experiences

**DOI:** 10.1111/cch.70126

**Published:** 2025-06-25

**Authors:** Heleen N. Haspels, Hennie Knoester, Faridi S. Jamaludin, Clara D. van Karnebeek, Mattijs W. Alsem

**Affiliations:** ^1^ Department of Pediatric Intensive Care Amsterdam UMC Location University of Amsterdam Amsterdam the Netherlands; ^2^ Amsterdam Reproduction & Development Research Institute Amsterdam the Netherlands; ^3^ Department of Neonatal & Pediatric Intensive Care, Division of Pediatric Intensive Care Erasmus Medical Center – Sophia Children's Hospital Rotterdam the Netherlands; ^4^ On behalf of the Transitional Care Unit Consortium Amsterdam the Netherlands; ^5^ Transitional Care Unit “Het Jeroen Pit Huis” Amsterdam the Netherlands; ^6^ Amsterdam UMC Location University of Amsterdam, Medical Library AMC Amsterdam the Netherlands; ^7^ Department of Pediatrics and Human Genetics, Emma Center for Personalized Medicine, Amsterdam Reproduction and Development Amsterdam UMC Location University of Amsterdam Amsterdam the Netherlands; ^8^ Department of Rehabilitation, Physical Therapy Science and Sports, UMC Utrecht Brain Center University Medical Center Utrecht Utrecht the Netherlands

**Keywords:** complex needs, review, transition

## Abstract

**Background:**

Hospital‐to‐home (H2H) transitions for children with medical complexity (CMC) are challenging and time‐intensive, often overwhelming parents due to insufficient care coordination, poor communication among healthcare professionals (HCPs), and limited family education. These shortcomings impact children, families and HCPs alike, highlighting the urgent need for improve H2H care for CMC. A key knowledge gap concerns HCP perspectives, as they play a pivotal role in ensuring care aligns with family needs.

**Objective:**

To synthesize HCP needs, views and experiences with H2H transitions for CMC and inform optimized care strategies.

**Eligibility Criteria:**

Eligible studies were peer‐reviewed original research on HCP experiences with H2H transitions for CMC, without restrictions on healthcare setting, publication year or study design.

**Data Sources:**

Systematic searches were conducted in MEDLINE, Embase, PsycINFO and CINAHL through July 2024.

**Data Extraction and Analysis:**

Two independent reviewers screened studies using predefined inclusion criteria. Study characteristics and HCP perspectives were extracted using a piloted form. Qualitative content analysis was used to synthesize HCP experiences. The resulting themes were organized using the socio‐ecological model, which describes elements at individual, interpersonal, organizational, community and societal levels.

**Results:**

Of 4087 identified records, 40 reports met eligibility criteria. Eleven themes were identified, with care coordination and continuity challenges spanning all socio‐ecological levels. Key challenges included HCP knowledge gaps and emotional burden (individual level), strained communication with parents and among HCPs (interpersonal level), inadequate care and educational plans (organizational level), restricted resources (community level) and bureaucratic hurdles (society level).

**Conclusion:**

This scoping review identifies multi‐level challenges HCPs face in supporting H2H transitions for CMC. The findings can guide the development of supportive interventions and healthcare innovations to strengthen care coordination, professional preparation, and cross‐setting collaboration. In addition, they provide direction for future targeted research.

**Summary:**

- This scoping review synthesizes healthcare professionals' experiences from 40 international studies, identifying 11 key themes structured within the socio‐ecological model. This offers a comprehensive framework to understand and improve hospital‐to‐home transitional care for children with medical complexity.
- The findings can guide the development of supportive interventions, including care coordination programs, structured education for families and professionals, and transitional care units.
- Further research is needed to evaluate the effectiveness, feasibility, and contextual adaptability of transitional care interventions, and to strengthen interprofessional collaboration across hospital and community settings.

**Abbreviations**
CMC: children with medical complexity
H2H: hospital to home
HCP: healthcare professional

## 1 Introduction

Chronically ill children navigate multiple transitions throughout the continuum of care, spanning different life stages—from birth to adulthood—and across various care settings and healthcare professionals (HCPs) (Brown 2018). These transitions during childhood include shifts such as moving from a primary care provider (PCP) to a subspecialist, PCP to emergency department (ED), ED to hospital and from hospital to home (H2H). Additionally, these children face the complex transition from paediatric to adult care as it often involves adjusting to new care teams, systems and expectations (Cassidy et al. 2022). While continuity is ideal, these transitions are inevitable and pose practical and organizational challenges, such as ensuring clear communication and managing timely transfer of medical information, for both patients and HCPs (Coleman and Boult 2003).

Care transitions are especially difficult for children with medical complexity (CMC)—defined as those with chronic conditions, technology dependency, high healthcare utilization and high family needs (Cohen et al. 2011; Millar et al. 2024). These patients and families face extensive, often lifelong care needs, involving repeated hospitalizations and frequent interactions with multiple HCPs and care systems (Rogers et al. 2021). H2H transitions, which occur frequently for CMC families, are acknowledged as particularly vulnerable periods (Breneol et al. 2017). Studies on parental experiences reveal it is often time‐intensive, overwhelming and fraught with challenges such as insufficient care coordination, poor communication among healthcare professionals, and inadequate education and training for families (van de Riet et al. 2023). Improving the organization of H2H care for CMC has become a prevalent issue, as children, their families and HCPs suffer from inadequate transitions homeward (Elias et al. 2012; Pordes et al. 2018; Auger et al. 2015).

To improve H2H care for CMC, it is essential to understand not only parental needs but also the needs, views and experiences of HCPs. Addressing these needs is crucial, as HCPs play a key role in supporting parents during the transition home. Although individual, often single‐center studies have examined these aspects, a comprehensive synthesis of this evidence is lacking. This scoping review aims to map these needs, views and experiences of HCP in providing H2H transitional care for CMC in clinical practice. The findings can inform the development of supportive interventions and healthcare improvements that better align with the needs of both CMC families and their providers, while also guiding future research priorities.

## 2 Methods

This scoping review of the literature was conducted in accordance with the Joanna Briggs Institute methodology for scoping reviews (Peters et al. 2020). The scoping review was chosen for its ability to capture various aspects of healthcare professionals experiences in transitional care, including differences across locations and professions while identifying knowledge gaps in this broad field (Crilly et al. 2010). The results are described based on the Preferred Reporting Items for Systematic Reviews and Meta‐Analyses extension for Scoping Reviews (PRISMA‐ScR) (Tricco et al. 2018). The study protocol was published on Open Science Framework (https://osf.io/ebg8h/).

### 2.1 Search Strategy and Inclusion Criteria

The databases of MEDLINE, Embase, PsycINFO and CINAHL were systematically searched in consultation with an information specialist (FJ). Inclusion criteria were studies that (1) examined the experiences, needs and views of HCPs on hospital‐toto‐home transitions for CMC, (2) peer‐reviewed original studies and (3) studies available in English and full text. Exclusion criteria were studies focusing on (1) transition of care from paediatric toward adult care, (2) solely on adult care and/or (3) needs, views and experiences of parents and families. Detailed search strategy is presented in Appendix S1. No limits were set on publication date, study design,or country of origin. The databases were searched on 15 July 2024. Duplicate articles were identified and deduplicated by using Endnote 20 (Gotschall 2021).

### 2.2 Study Selection

Study selection was carried out using the web application Rayyan (Ouzzani et al. 2016). Two independent reviewers (HH/HK or HH/MA) assessed all papers for eligibility through initial screening of titles and abstracts. Subsequently, the two reviewers independently studied the full texts for final inclusion. Snowball sampling by searching the reference lists of all included articles was done to identify additional publications. When disagreement needed to be resolved, a third reviewer was consulted.

### 2.3 Data Extraction

Data were extracted by one reviewer (HH) and verified by a second independent reviewer (HK/MA). A self‐designed extraction form was used which was tested in three articles by three researchers (HH/HK/MA) before its official implementation. Extracted data include author, year of publication, country of origin, study purpose, participants, methods of data collection, data analysis and key findings. In articles where both HCP and parental experiences were described, only data from HCP were extracted. When the focus of the article extended beyond H2H transitional care within CMC care, only the key findings related to transitional care were extracted.

### 2.4 Data Analysis

Results were analysed based upon on the latest recommendations of the JBI Scoping Review Methodology Group (Pollock et al. 2023). Quantitative data were converted into textual data through repeated detailed examination based on the research questions (Lizarondo et al. 2019; Stern et al. 2021). This was combined with data from qualitative studies, enabling integration of qualitative and quantitative findings. A qualitative content analysis using an inductive approach was undertaken to analyse qualitative data (Elo and Kyngäs 2008). This consisted of three phases: (i) preparation, (ii) organization and (iii) reporting. In the preparation phase, a qualitative analysis using an inductive approach was identified as suitable for this review given the purpose to map the peer‐reviewed literature about a topic on which there is dearth of evidence. During the organization phase, two independent authors (HH/HK or HH/MA) collaboratively performed open coding, developed categories, and identified overarching themes. The socio‐ecological model (SEM), originally developed by Bronfenbrenner (Bronfenbrenner 1979; Bronfenbrenner 1977), was adapted as a framework to analyse and present the findings (Control CfD, Prevention 2016). This framework was identified during the analysis as particularly suitable for capturing the multifaceted interactions between individuals and their environment. It allowed for the categorization of themes across the individual, interpersonal, institutional, community and society levels. Last, the reporting phase involved a narrative summary of the themes which emerged during the previous stages. Analysis was facilitated by qualitative data analysis software (MAXQDA).

## 3 Results

### 3.1 Search Results

**FIGURE 1:**
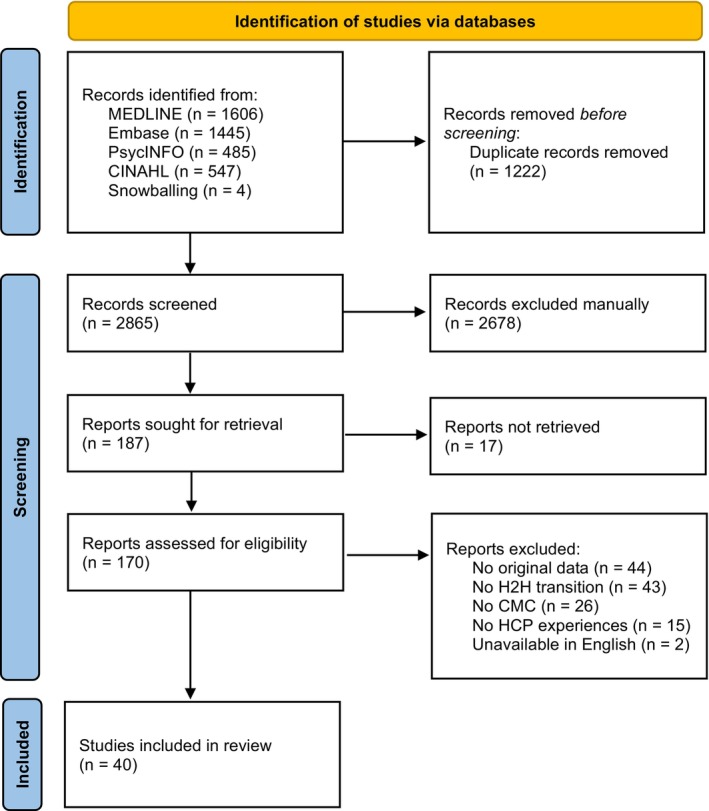
PRISMA flow diagram.

A total of 4087 records were identified through database searching. After removing duplicates (*n* = 1222) and excluding non‐relevant studies by screening titles and abstracts (*n* = 2678), 187 reports were sought for a full text assessment. Of these, 40 reports—each representing a unique study—were included in this review (Figure 1) (Abbott et al. 2005; Abebe et al. 2020; Adams et al. 2013; Babayan et al. 2023; Barnard et al. 2013; Brenner et al. 2018a; Brenner et al. 2018b; Brenner et al. 2020; Carter et al. 2016; Coller et al. 2017; Coller et al. 2020; Cuevas‐Asturias et al. 2024; Curran et al. 2020; Dallas et al. 2023; Fratantoni et al. 2019; Glick et al. 2024; Gorsky et al. 2023; Gupta et al. 2004; Henderson et al. 2017; Kirk 1999; Kirk 2001; Kirk and Glendinning 2002; Kirk and Glendinning 2004; Kobussen et al. 2020; Law et al. 2011; Leyenaar et al. 2017; Leyenaar et al. 2018; Manhas and Mitchell 2012; Manhas and Mitchell 2015; McLorie et al. 2023; Nageswaran et al. 2020; Nelson et al. 2023; Noyes et al. 2014; Price et al. 2018; Ramalho et al. 2022; Ravid et al. 2020; Sobotka et al. 2020; Tearl et al. 2006; Tearl and Hertzog 2007; van de Riet et al. 2024).

### 3.2 Study Characteristics

The largest proportion of the 40 studies were conducted in the United States (*n* = 17, 42.5%), followed by the United Kingdom (*n* = 9, 22.5%) and Canada (*n* = 6, 15%) (Table 1). Most studies utilized a qualitative study design (*n* = 23, 57.5%), followed by mixed‐method studies (*n* = 14, 35%), with a smaller number of quantitative studies (*n* = 3, 7.5%). Most studies were conducted in (academic) hospital settings (*n* = 17, 43.6%), followed by studies that included both hospital and community settings (*n* = 16, 41.0%). The studies captured the experiences of a median of 31 healthcare professionals (range 5–803) from a wide range of disciplines. Nurses (*n* = 28, 77.8%) and physicians (*n* = 28, 77.8%) were the most frequently represented professional groups (Table 1). Other groups, such as social workers (*n* = 13, 36.1%), allied healthcare professionals (*n* = 11, 30.6%) and care coordinators (*n* = 11, 30.6%), were also commonly included, highlighting the multidisciplinary nature of care for CMC. More details of the included studies are provided in Appendix S2.

**TABLE 1:** Characteristics of included studies.

	Total *n* = 40
**Country**	
United States	17 (42.5)
United States and Canada	1 (2.5)
Canada	6 (15)
United Kingdom	9 (22.5)
Europe	3 (7.5)
Brazil	1 (2.5)
Ireland	1 (2.5)
Netherlands	1 (2.5)
Scotland	1 (2.5)
**Study methods**	
Qualitative	23 (57.5)
Mixed method	14 (35)
Quantitative	3 (7.5)
**Number of healthcare professionals** ^**a**^	31 (5–803)
**Type of HCP** ^**b**^	
(Specialized) nurses	28 (77.8)
Paediatricians/medical specialists	28 (77.8)
Allied healthcare professionals	11 (30.6)
Case managers/care coordinators	11 (30.6)
Psychologists	4 (11.1)
Social workers	13 (36.1)
Administrative staff, clinical directors and/or managers	8 (22.2)
Others	12 (33.3)
**Work setting HCP** ^**c**^	
(Academic) hospital	17 (43.6)
Hospital and community	16 (41)
Hospital and ambulatory	1 (2.6)
Home care	1 (2.6)
Multi‐agency services	1 (2.6)
Members of AAP	1 (2.6)
Inpatient, hospital‐based outpatient and community‐based	1 (2.6)

*Note:* Data are *n* (%) or median (range).

^a^ Of 34 studies (6 studies did not reported number of HCPs).

^b^ Of 36 studies (4 studies did not reported number of HCPs).

^c^ Of 39 studies (1 study did not reported work setting of HCPs).

### 3.3 HCP Experiences

**FIGURE 2:**
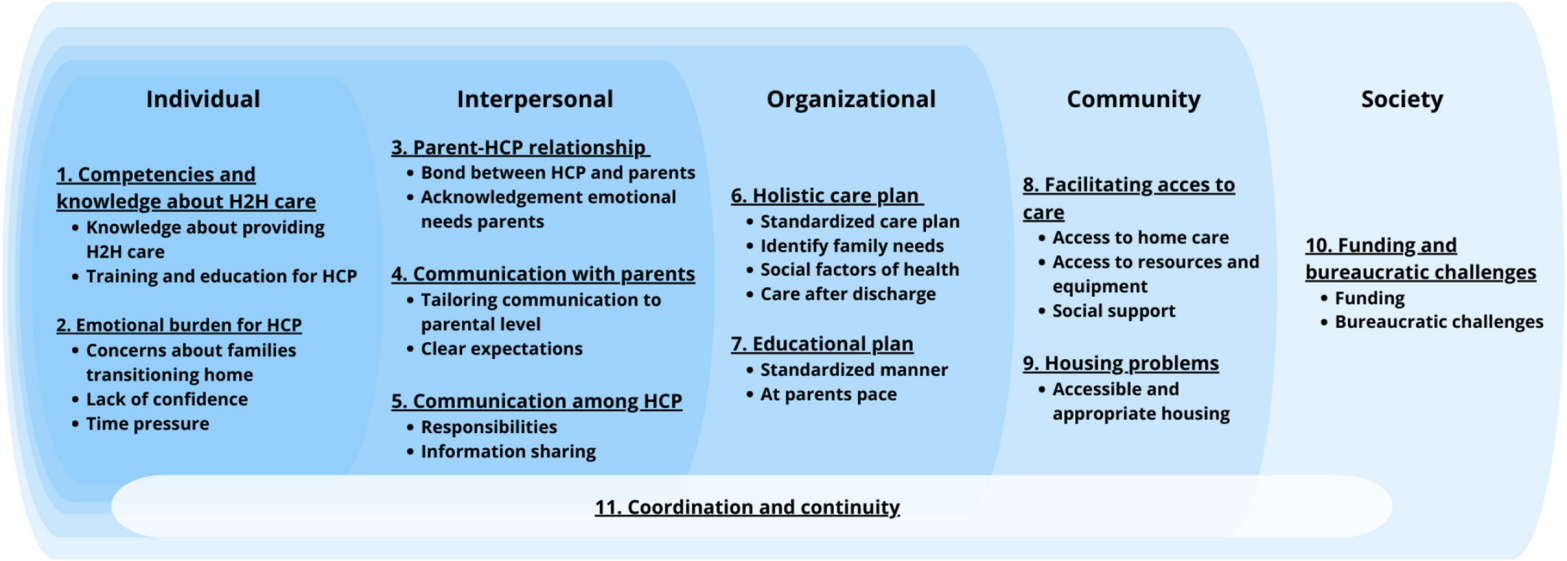
The 11 identified themes with categories across the social‐ecological model.

From the 40 included articles, 450 codes were extracted, capturing the HCP experiences of H2H transitions. These codes were organized into 24 categories, which were ultimately consolidated into 11 overarching themes. The 11 themes and their categories within the social‐ecological model are presented in Figure 2 and discussed below. An overview of identified themes, categories and a selection of open coding illustrated by citations is presented in Appendix S3. Additionally, Appendix S4 illustrated the distribution of the 11 themes across the 40 included papers.

### 3.4 Individual Level

#### 3.4.1 Theme 1: Competencies and Knowledge About H2H Care

At the individual level, HCPs reported that providing H2H care is a specialized field requiring specific competencies and knowledge. Some report limited knowledge of arranging appropriate home care and the realities of life at home for patients and families, hindering their ability to provide tailored support. Additionally, many are unaware of existing initiatives to support families, particularly those with social vulnerabilities. HCPs emphasized the need for targeted training and education in H2H care to improve this awareness and preparedness. A participant in Henderson (2017) described this knowledge gap as a systemic issue: ‘[ICU staff] discharge kids without adequate support. They don't have a vision of what their lives are like… They need the insight; they need to be familiar with really what happens’.

#### 3.4.2 Theme 2: Emotional Burden for HCP

Another significant theme at the individual level is the emotional burden experienced by HCPs in providing H2H care. This burden, as reported by HCPs, stems from their awareness of the complex challenges families (including siblings) face, and their concerns about how adequately families are prepared to transition from 24/7 hospital care to life at home. Many HCPs report feeling a lack of confidence in their ability to provide sufficient support during this transition. These emotional challenges are further exacerbated by external pressures, such as the need to free up hospital beds and manage overwhelming workloads—issues reported by both hospital and home care professionals. As one professional described: ‘We're under increasing pressure from the hospital side about blocking beds, needing to move, winter's coming in, infections are rife, and it's move, move, move. So, we're being propelled and driven along a road at a rate that… the two things are at odds necessarily with each other’ (Price et al. 2018).

### 3.5 Interpersonal Level

#### 3.5.1 Theme 3: Parent–HCP Relationship

HCPs emphasized that strong interpersonal connections with parents are crucial for delivering high‐quality H2H care. Continuity in these relationships was particularly valued, as it helped ‘pull things together’ for families. As described in one study, ‘The nurses talked about how they “pulled things together” for families by dedicating time to build in‐depth relationships with the families and the stakeholders across a variety of settings’ (Carter et al. 2016). Across studies, HCPs described parents as partners in the care process, recognizing not only their emotional needs, but also their unique expertise regarding their child's condition, daily routines, family context and available community resources. This acknowledgment of parental knowledge and strengths was seen as essential for effective collaboration and shared decision‐making. At the same time, HCPs highlighted the need to balance empathy with professionalism, as excessive emotional involvement can blur the boundaries of the professional relationship.

#### 3.5.2 Theme 4: Communication With Parents

Effective communication with parents was described by HCP's as essential to ensuring that parents fully understand the information provided. Professionals reported the need to tailor communication to the parents' level, taking into account factors such as language barriers and difficulties in processing complex information. Beyond the content, HCPs emphasized the value of using multiple formats to enhance comprehension. As one professional put it: ‘People learn information in multiple different ways … I always have a written or visual component, ideally with pictures … in the primary language of the patient, and then I also verbally go over it … It can't just be spoken word’ (Glick et al. 2024). A key aspect of communication is managing parental expectations, helping them navigate their child's immediate needs and long‐term development. However, HCPs perceive that uncertainty about an infant's health trajectory and inconsistent updates can cause significant frustration for parents.

#### 3.5.3 Theme 5: Communication Among HCP

Communication challenges among HCPs were commonly reported during transition periods, primarily due to the involvement of multiple providers. HCPs described unclear delineation of roles and confusion about ultimate responsibility for a child's care, which often led to frustration. As one study noted, ‘Even professionals themselves were sometimes unclear about their responsibilities; both GPs and nurses were unsure whether GPs or hospital consultants were medically responsible for children while at home’ (Kirk and Glendinning 2004). The shifting of responsibilities during transitions was seen as complicating coordination efforts and reinforcing the need for consistent and structured communication. Sharing medical information also presented difficulties, as HCPs noted that data transfer between care settings was frequently inefficient, and maintaining up‐to‐date information remained a challenge.

### 3.6 Organizational Level

#### 3.6.1 Theme 6: Holistic Care Plan

At the organizational level, HCPs stress the importance of developing a holistic care plan to support successful H2H transitions. According to HCPs, such plans ideally balance standardization and individualization: while core components of transitional care were seen as broadly applicable across cases, HCPs emphasized the need to tailor interventions to the unique needs of each child and family. Care plans were reported to be most effective when rooted in family needs and developed through listening, collaboration, and shared decision‐making. In several studies, HCPs noted that effective care planning also required attention to broader social factors, including the home environment, financial challenges, and the availability of social support. HCPs believe addressing these aspects during hospitalization helps establish a stable foundation for families after discharge. For example, some HCPs described arranging respite care or connecting families with support systems as helpful in easing the burden on families. Additionally, HCPs reported that elements of post‐discharge care—such as follow‐up appointments, phone calls shortly after discharge, and the use of telemedicine—need to be arranged during the hospital stay to ensure continuity of care. As one professional emphasized: ‘Follow up with these chronic families. Make a call the next day … Just to make them feel like you know … we're still connected. I'm not doing this alone. I think that makes all the difference in the world’ (Leyenaar et al. 2017).

#### 3.6.2 Theme 7: Educational Plan

At the organizational level, HCPs feel responsible for equipping parents with the knowledge and skills required to manage their child's care at home. Across studies, professionals described a preference for a standardized educational plan, focusing on both practical caregiving skills and broader empowerment to help parents feel confident and prepared for their caregiving role. One professional remarked: ‘Some [families] are over‐taught and some of them are briefed and then sent out the door. I think if the process was the same for all families, it would be great’ (Nageswaran et al. 2020). Several HCPs emphasized the importance of allowing parents to learn step by step and at their own pace, noting that trust in parents' abilities tends to grow gradually through the process. The educational trajectory was often described as lengthy, with professionals highlighting the value of celebrating small milestones to motivate and support families. Key components of training included managing emergencies, pain and symptom control, and promoting a sense of self‐efficacy among parents. At the same time, HCPs noted challenges when parents were unavailable to consistently participate in training sessions. In these cases, some professionals questioned whether the hospital setting was the most appropriate place for continued preparation, particularly for children who were medically stable. A number of HCPs suggested that step‐down units might offer a more suitable environment for ongoing parent education and care planning.

### 3.7 Community Level

#### 3.7.1 Theme 8: Facilitating Access to Care

At the community level, HCPs reported significant challenges in ensuring access to adequate care for families at home, particularly due to difficulties in recruiting and retaining qualified healthcare personnel, obtaining appropriate medical equipment, and securing social support services. These issues were described as contributing factors to prolonged hospital stays or even readmissions. Professionals emphasized the importance of knowing how to arrange these services, but noted that access to care and social support varied greatly between communities. Delays in acquiring medical equipment for home use and inconsistencies between hospital and home‐based systems were frequently mentioned. ‘Sometimes we're sending medications out into the void’, one provider noted, ‘not knowing if parents are actually going to get these medications … or if the pharmacy even has the medicine in the right form’ (Glick et al. 2024).

In addition, HCPs observed that families often experienced financial hardships and lacked access to essential resources such as social work support to help navigate complex care arrangements. According to several HCPs, this lack of community‐level support placed additional strain on families caring for medically complex children at home.

#### 3.7.2 Theme 9: Housing Problems

HCPs identified challenges related to housing as a significant barrier to effective H2H care at the community level. Families were often reported to live in homes that were not suited to the medical and safety needs of their children. HCPs described that appropriate housing needed to be both accessible and adaptable for medical equipment, but such housing options were often unavailable or difficult to obtain in many municipalities. As one provider noted: ‘These are families that are complex in the nature of potentially being homeless, not having appropriate accommodation. It's housing that takes ages because if there's no houses, there's no houses. There's nowhere to discharge this child’ (McLorie et al. 2023). HCPs also noted that navigating the procedures to secure appropriate housing was frequently a lengthy and frustrating process, which added to the stress experienced by families.

### 3.8 Society Level

#### 3.8.1 Theme 10: Funding and Bureaucratic Challenges

At the societal level, HCPs reported that challenges related to funding structures and bureaucratic procedures significantly hindered effective H2H transitions. One recurring issue involved uncertainty around who was responsible for funding specific types of care. As noted in one study, ‘it is unclear where funding responsibilities for short‐term care lie, leading to inconsistent interpretations at the local level and variations in the services available to families’ (Kirk 1999). HCPs described bureaucratic hurdles as contributing to delays and frustrations, with rigid administrative processes making it difficult to arrange timely and appropriate care. Differences in equipment requirements between home and hospital settings, often dictated by insurance policies, lead to substantial delays in discharging patients.

### 3.9 Integrative Theme

#### 3.9.1 Theme 11: Care Coordination and Continuity

The complexity of care regimens for CMC was consistently reported by HCPs as a major challenge across all levels of the socio‐ecological model. HCPs emphasize that effective care coordination and continuity are essential components of H2H care for CMC. At the individual and interpersonal level, HCPs described the importance of taking an active role in coordinating care and ensuring clarity in communication. They emphasized the need for clearly defined roles and responsibilities, and highlighted the value of maintaining alignment with parents to support shared understanding and collaboration. At the organizational and community level, HCPs noted that care coordination extended beyond the hospital setting, requiring alignment with a range of stakeholders including outpatient clinics, primary care providers, pharmacies, and social services. Several HCPs stressed the importance of continuity of care across institutional boundaries to prevent fragmentation and ensure seamless transitions for families. When this coordination is lacking, the burden often falls on families. One study reported: ‘When prescribers lack care‐coordination support or experience, families become case managers by default, responsible for identifying home health needs and investigating options’ (Fratantoni et al. 2019).

### 3.10 Proposed Interventions

**FIGURE 3:**
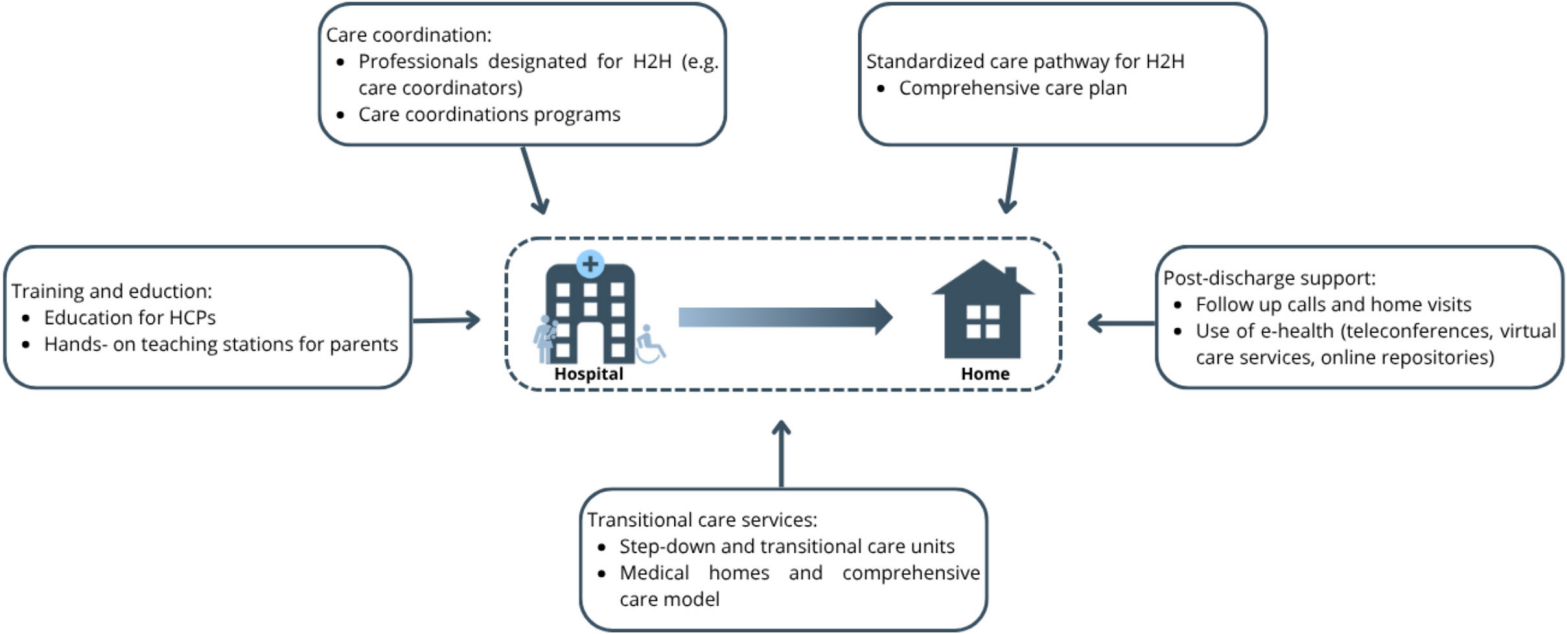
Interventions to improve H2H care proposed in the included studies.

In addition to the experiences of HCPs, the included articles highlighted specific interventions to improve H2H transitions for CMC. Key interventions included professionals designated for H2H transitions, care coordination programs, training and education for parents and HCPs, standardized care pathways, transitional care services such as step‐down units, and the use of telehealth services and home visits to provide post‐discharge support (Figure 3).

## 4 Discussion

This scoping review aimed to explore and synthesize the experiences of HCPs in the H2H transition for CMC. Through the analysis of 40 studies, we identified 11 themes spanning individual, interpersonal, organizational, community and societal levels that influence these transitions. These themes are (1) competencies and knowledge about H2H care, (2) emotional burden for HCPs, (3) parent–HCP relationship, (4) communication with parents, (5) communication among HCPs, (6) holistic care plans, (7) educational plans, (8) facilitating access to care, (9) housing problems, (10) funding and bureaucratic challenges and (11) care coordination and continuity. Collectively, these themes underscore the complex, multi‐level challenges HCPs encounter in supporting H2H transitions.

While the literature predominantly focuses on improving outcomes for children and their families during H2H transitions, our findings prompt reflection on whether greater attention should be directed toward HCPs as a critical enabler of high‐quality care. Across studies, HCPs reported lacking the necessary competencies, confidence, and structural support to provide effective family‐centered transitional care. These gaps may hinder their ability to adequately prepare families and to coordinate care across settings. Despite these reported needs, the literature offers limited evidence on structured training or capacity‐building interventions for HCPs. Noyes et al., underscores this gap by highlighting that current discharge processes often lack essential system‐level components—such as communication loops, audit and feedback mechanisms, and shared frameworks across care levels—that would allow for adaptive learning and improvement (Noyes et al. 2014). The authors propose a systems‐theoretical model to conceptualize the transition process as a complex intervention, and stress the need for future research to develop and evaluate interventions that effectively support professionals in their roles. These findings suggest that strengthening transitional care requires not only attention to the needs of children and families but also investment in the systems, structures and capacities that enable professionals to deliver responsive and coordinated care.

Improving HCP confidence and skills not only strengthens professional performance but also contributes to more trusting and collaborative relationships with families. Indeed, the importance of the parent–HCP relationship emerged as a key theme in our review, with professionals emphasizing the value of strong interpersonal connections in navigating complex care trajectories. This is echoed in previous research, which highlights the critical role of the parent–child–HCP triad in supporting families of CMC (Golden and Nageswaran 2012; Miller et al. 2009; Adams et al. 2017). Trusting relationships with HCPs have been shown to enhance parental empowerment and credibility, both of which are vital for navigating the complexities of home care (Adams et al. 2013). Together, these insights emphasize the need to better understand and support the professional side of transitional care as an integral part of improving outcomes for families.

### 4.1 Healthcare Professional vs Parental Experiences

The findings from HCPs reveal several parallels with the qualitative experiences of parents of CMC as aggregated by van de Riet et al. (2023). Both groups emphasize the critical importance of care coordination and continuity, highlighting the necessity of a comprehensive and family‐centered care plan to facilitate successful H2H transitions. Effective communication is prioritized by both parents and professionals, with overlapping and distinct emphases. Both groups underscore the importance of accessible and consistent information tailored to the needs of families. Parents specifically highlight the value of receiving information in clear, understandable language and emphasize the need for consistency to build their confidence as caregivers. Professionals, in addition to recognizing these needs, also stress the importance of clear communication within multidisciplinary teams to ensure cohesive and coordinated care delivery. This shared focus on communication highlights its pivotal role in facilitating successful H2H transitions. Training and preparation are likewise seen as essential. Parents often value practical, tailored instruction, whereas professionals advocate for standardized educational programs with structured modules or hands‐on teaching sessions for common technical tasks (Coller et al. 2017). Emotional challenges are another point of overlap: parents describe feelings of anxiety and vulnerability, while professionals report the emotional burden of supporting families in complex and uncertain situations. Despite these parallels, important differences emerge. Parents tend to prioritize flexible, personalized approaches to training and strongly value being acknowledged as experts in their child's care. In contrast, professionals may favor more standardized protocols and often face the challenge of balancing empathy with professional distance. These differences underline the need for transitional care interventions that are co‐designed with families and that reflect both professional perspectives and lived family experience.

### 4.2 Intervention Research

Research into interventions supporting the H2H transition for CMC has predominantly focused on improving care coordination, communication and multidisciplinary collaboration (Breneol et al. 2017; de Lange et al. 2023). These areas were also emphasized by healthcare professionals in our review, particularly in relation to the need for clearly defined roles (e.g., care coordinators) and structured care coordination programs. While evidence suggests that complex care programs can reduce healthcare costs and improve parental satisfaction (Gordon et al. 2007; Cohen et al. 2010; Casey et al. 2011), these findings are often based on evaluations of hospital‐initiated programs. Although some of these initiatives involve collaboration with primary care providers, there is a lack of interprofessional studies that examine how cross‐boundary collaboration between hospital and community‐based providers can be effectively structured and sustained.

Training and education for both families and HCPs were frequently proposed as essential interventions. Family‐centered discharge education has been associated with improved patient outcomes and reductions in potentially avoidable healthcare utilization (Desai et al. 2015). Simulation‐based training programs have demonstrated their effectiveness in improving parents' clinical skills, comfort and confidence in managing their child's care, including in emergency situations (Thrasher et al. 2018; Tofil et al. 2013; Whalen et al. 2020; Yuen et al. 2021). As HCP's feel responsible for adequate and tailormade education before being convinced that parents are able to take care of their child in the home situation, research of training and education programs is essential to improve care for CMC and their families.

Reorganizing care delivery through transitional care services, such as medical homes and transitional care units, has also been proposed as a promising approach. Studies have demonstrated that children with tracheostomies spend fewer days in acute care hospitals when post‐acute care facilities are utilized (Feudtner et al. 2005; Berry et al. 2009). Additionally, transitioning to a home‐like step‐down care facility has been shown to provide significant benefits for both parents and children, allowing them to ‘live again’ instead of merely ‘exist’ (Price et al. 2018). Transitional care services may also offer advantages for HCPs, who benefit from working in a specialized setting where the entire team is focused on transitional care for CMC.

### 4.3 Cross‐National Variation

This review included studies from various healthcare systems, such as those in the United States, United Kingdom and Canada. While these systems differ greatly in terms of organization, funding, and care coordination, many of the challenges reported by HCPs were remarkably similar across countries. Even within individual countries, the organization of H2H care can vary regionally, making it difficult to link experiences to specific system designs. Most included studies also lacked detailed contextual information about local healthcare structures, which limits the ability to draw meaningful cross‐national comparisons. Nevertheless, the consistent emergence of key themes suggests that these challenges are shared across diverse settings. The findings, therefore, provide broadly relevant insights for improving H2H care internationally.

### 4.4 Strengths and Limitations

This study has several notable strengths, including capturing the perspectives of a wide range of HCPs, including nurses, physicians, social workers and allied healthcare professionals, highlighting the multidisciplinary nature of H2H care for CMC. Additionally, the inclusion of HCPs from various settings, such as hospitals and community‐based care, provides a comprehensive understanding of H2H transitions across the continuum of care. Finally, the use of qualitative, quantitative and mixed‐method studies enhances the robustness of the analysis and offers a well‐rounded perspective on HCP experiences. However, several limitations should be noted. Most of the studies were conducted in high‐income countries such as the United States, Canada and Europe, which may limit the generalizability of findings to low‐ and middle‐income countries. Moreover, while this scoping review provided a broad overview of available evidence, a qualitative evidence synthesis with meta‐analytic approach may have yielded deeper interpretative insights into the experiences of HCPs. At the outset, we did not anticipate such a large proportion of qualitative studies to be available, which may warrant a follow‐up synthesis focused specifically on qualitative findings.

### 4.5 Future Perspectives

While the included studies offer important insights, they also reveal critical knowledge gaps that warrant further exploration. First, there is a need for interprofessional and cross‐sectoral research that examines how collaboration between hospital and community‐based providers can be effectively structured and sustained. Despite frequent references to the importance of care coordination, few studies investigate how such collaboration is implemented in practice or how it can be supported over time. Second, although several interventions were proposed—such as care coordination programs, family education, and telehealth follow‐up—there is limited evidence on their effectiveness, feasibility and contextual adaptability. Future research should focus on the development, implementation and evaluation of transitional care interventions, using theoretical frameworks that account for the complexity of care systems. Third, the literature rarely includes detailed contextual information about healthcare settings, policies or organizational factors. This limits the ability to draw conclusions about what works, where, and under which conditions. To advance the field, comparative research across regions and countries is needed, and we encourage healthcare professionals to build collaborative networks that can facilitate such multi‐center and cross‐national studies. Finally, most studies approach transitional care from the perspective of individual professionals or organizations, while system‐level dynamics—such as funding structures, feedback loops, and accountability mechanisms—remain underexplored. Future work could benefit from using systems‐thinking approaches to better understand how professionals operate within and across healthcare boundaries. Importantly, such approaches should actively involve patients and families in co‐shaping system improvements, ensuring their lived experiences inform policy, design, and delivery at every level. By addressing these gaps, future research can move beyond description toward the development of practical, evidence‐informed strategies that support both healthcare professionals and families in navigating complex transitions.

### 4.6 Conclusion

This scoping review underscores the multi‐level challenges healthcare professionals face in facilitating H2H transitions for CMC. While valuable insights exist, important gaps remain regarding interprofessional collaboration, the evaluation of interventions and system‐level support structures. The current evidence base calls for targeted research to identify which care components are essential, for whom, and under what conditions. Such insights will be critical in informing the development of evidence‐based, adaptable models for transitional care. Addressing these gaps through context‐sensitive and systems‐informed research will be essential for strengthening transitional care practices and improving outcomes for children, families, and the professionals who support them.

## Author Contributions

**Heleen N. Haspels:** conceptualization, methodology, formal analysis, investigation, writing – original draft, visualization. **Hennie Knoester:** conceptualization, methodology, formal analysis, investigation, writing – review and editing. **Faridi S. Jamaludin:** methodology, resources, writing – review and editing. **Clara D. van Karnebeek:** conceptualization, methodology, writing – review and editing, funding acquisition. **Mattijs W. Alsem:** conceptualization, methodology, formal analysis, writing – review and editing, supervision. All authors approved the final manuscript as submitted and agree to be accountable for all aspects of the work.

## Ethics Statement

Ethics approval was not required for this scoping review, as it involved analysis of previously published literature only.

## Consent

This study did not involve human participants or personal data.

## Conflicts of Interest

The authors declare no conflicts of interest.

## Supporting information


**Appendix S1** Detailed search strategy.
**Appendix S2** Characteristics of included studies.
**Appendix S3** Overview of identified themes, categories and a selection of open coding illustrated by citations.

## Data Availability

The data that support the findings of this study are available from the corresponding author upon reasonable request.
