# Supplementary material for: Hospital to Home Transitions for Children With Medical Complexity: A Scoping Review of Healthcare Professionals Experiences
Source: Child Care Health Dev. 2025 Jun 25;51(4):e70126. doi: 10.1111/cch.70126 (PMC12196559; doi:10.1111/cch.70126)
Supplement: Supplementary file 1 — Appendix S1 Detailed search strategy. Appendix S2 Characteristics of included studies. Appendix S3 Overview of identified themes, categories and a selection of open coding illustrated by citations. [file CCH-51-e70126-s001.docx]

**Appendix 1: Detailed search strategy**

The population, concept and context (PCC) framework will be used as the criteria for identifying studies (Table 1).

Table 1 PCC framework

| Population | This study will include healthcare professionals who are involved in the care during transitions from hospital to home for CMC across different health settings and specialties, including hospital, hospice and home settings. CMC as defined by Cohen et al as children with one or more complex chronic illness that require specialized care; have functional disabilities; high healthcare utilization; and high family-identified needs [1]. |
| --- | --- |
| Concept | The concept of interest is the needs, views and experiences of HCPs caring for CMC during the transition of care from a hospital setting towards home. |
| Context | This review will consider any study that has been performed within a healthcare system that provides both hospital care and complex home care. |
| Types of evidence sources | This review will consider relevant qualitative, quantitative and mixed methods studies for inclusion. |

MEDLINE (OVID):

Database(s): Ovid MEDLINE(R) ALL

Search Strategy:

| # | Searches |
| --- | --- |
| 1 | Transitional Care/ or Patient Transfer/ or Patient Discharge/ or ambulatory care/ or exp "continuity of patient care"/ or hospital to home transition/ |
| 2 | exp Child Health Services/ and (Tertiary Care Centers/ or Rehabilitation Centers/) |
| 3 | (transition* or discharge or co-ordinated service* or coordinated service* or pediatric care coordinat* or paediatric care coordinat*).ti,ab,kf. |
| 4 | (transfer* adj2 (care* or patient)).ti,ab,kf. |
| 5 | 1 or 2 or 3 or 4 |
| 6 | exp Home Care Services/ or Hospice Care/ |
| 7 | (medical home* or home care or unit* or clinic or service* or program* or framework* or model or hospice).ti,ab,kf. or home.ti. |
| 8 | ((hospital or transition* or discharge or nicu or picu) adj3 home).ti,ab,kf. |
| 9 | 6 or 7 or 8 |
| 10 | Needs Assessment/ or "Health Services Needs and Demand"/ or Educational Status/ or exp Self Care/ or self efficacy/ or "attitude of health personnel"/ or psychosocial functioning/ or psychological phenomena/ or perception/ or "behavior and behavior mechanisms"/ or exp adaptation, psychological/ or defense mechanisms/ or exp emotions/ or empowerment/ or help-seeking behavior/ or exp self-control/ or exp motivation/ or exp power, psychological/ or exp morale/ or psychological safety/ or psychosocial functioning/ or (education or psychology).fs. |
| 11 | (need* or challeng* or prefer* or wish* or obstacle* or worry or worries or confidence or experienc* or satisf* or empower* or self-management or education or perception* or view*).ti,ab,kf. |
| 12 | 10 or 11 |
| 13 | exp Health Personnel/ |
| 14 | (healthcare provider* or health care provider* or health personnel or medical personnel or medical staff or nurs* or doctor* or physician* or medical specialist* or clinician* or hospital-based clinician* or healthcare worker* or health care worker* or healthcare professional* or health care professional* or pediatrician* or paediatrician* or health administrator* or hospital personnel or hospital staff or health caregiver* or healthcare giver* or social worker* or psychosocial worker* or psychologist* or allied health personnel* or speech therapist* or rehabilitation physician* or physical therapist* or home nurse* or caregiver*).ti,ab,kf. |
| 15 | 13 or 14 |
| 16 | Disabled Children/ |
| 17 | (chronic* or medical complex* or medically complex* or comorbid* or co-morbid* or multiple morbidit* or complex medical need* or complex medical condition* or special medical condition* or complex health condition* or complex need* or complex care* or complex care need* or complex health need* or complex healthcare need* or complex health care need* or CSHCN or CSHCNs or CMC or yshcn* or disabled child* or medical fragil* or resource intensive need* or extensive health care need* or technology dependen* or complex technological health care need* or complex technological healthcare need* or special health care need* or complex multidisciplinary health*).ti,ab,kf. |
| 18 | ((medical* or need* or health condition* or care or disease*) adj3 complex*).ti,ab,kf. |
| 19 | ((special or extensive) adj3 need*).ti,ab,kf. |
| 20 | (neonatal intensive care unit* and transit*).ti,ab,kf. |
| 21 | 16 or 17 or 18 or 19 or 20 |
| 22 | exp Pediatrics/ or exp Child/ or exp Infant/ or Adolescent/ |
| 23 | (child* or infan* or neonat* or newborn* or baby or babies or preschool or preteen* or schoolchild* or pediatric* or paediatric* or juvenile or toddler* or kids or girl* or boy* or school age* or minors or adolescent* or teen* or youth).ti,ab,kf. |
| 24 | 22 or 23 |
| 25 | 5 and 9 and 12 and 15 and 21 and 24 |
| 26 | Transition to Adult Care/ or ((adult healthcare or adult health care or adult care or adult service* or young adult* or adulthood) not parent*).ti,ab,kw. or (transition to adult care or transition from child to adult health service* or transition from pediatric to adult care).ti,ab,kf. |
| 27 | 25 not 26 |

EMBASE (OVID):

Database(s): Embase Classic+Embase 
Search Strategy:

| # | Searches |
| --- | --- |
| 1 | transitional care/ or patient transport/ or hospital discharge/ or hospital to home transition/ |
| 2 | child health care/ and (tertiary health care/ or tertiary care center/ or rehabilitation center/) |
| 3 | (transition* or discharge or co-ordinated service* or coordinated service* or pediatric care coordinat* or paediatric care coordinat*).ti,ab,kf. |
| 4 | (transfer* adj2 (care* or patient)).ti,ab,kf. |
| 5 | 1 or 2 or 3 or 4 |
| 6 | exp health care delivery/ or hospice care/ |
| 7 | (medical home* or home care or unit* or clinic or service* or program* or framework* or model or hospice).ti,ab,kf. or home.ti. |
| 8 | ((hospital or transition* or discharge or nicu or picu) adj3 home).ti,ab,kf. |
| 9 | 6 or 7 or 8 |
| 10 | needs assessment/ or perception/ or personal experience/ or personal needs/ or self care/ or self care education/ or educational status/ or self help/ or exp self concept/ or exp emotion/ or empowerment/ or exp health personnel attitude/ or exp motivation/ |
| 11 | (need* or challeng* or prefer* or wish* or obstacle* or worry or worries or confidence or experienc* or satisf* or empower* or self-management or education or perception* or view*).ti,ab,kf. |
| 12 | 10 or 11 |
| 13 | exp Health Personnel/ |
| 14 | (healthcare provider* or health care provider* or health personnel or medical personnel or medical staff or nurs* or doctor* or physician* or medical specialist* or clinician* or hospital-based clinician* or healthcare worker* or health care worker* or healthcare professional* or health care professional* or pediatrician* or paediatrician* or health administrator* or hospital personnel or hospital staff or health caregiver* or healthcare giver* or social worker* or psychosocial worker* or psychologist* or allied health personnel* or speech therapist* or rehabilitation physician* or physical therapist* or home nurse* or caregiver*).ti,ab,kf. |
| 15 | 13 or 14 |
| 16 | Disabled Children/ |
| 17 | (chronic* or medical complex* or medically complex* or comorbid* or co-morbid* or multiple morbidit* or complex medical need* or complex medical condition* or special medical condition* or complex health condition* or complex need* or complex care* or complex care need* or complex health need* or complex healthcare need* or complex health care need* or CSHCN or CSHCNs or CMC or yshcn* or disabled child* or medical fragil* or resource intensive need* or extensive health care need* or technology dependen* or complex technological health care need* or complex technological healthcare need* or special health care need* or complex multidisciplinary health*).ti,ab,kf. |
| 18 | ((medical* or need* or health condition* or care or disease*) adj3 complex*).ti,ab,kf. |
| 19 | ((special or extensive) adj3 need*).ti,ab,kf. |
| 20 | (neonatal intensive care unit* and transit*).ti,ab,kf. |
| 21 | 16 or 17 or 18 or 19 or 20 |
| 22 | exp Pediatrics/ or exp Child/ or exp Infant/ or adolescent/ |
| 23 | (child* or infan* or neonat* or newborn* or baby or babies or preschool or preteen* or schoolchild* or pediatric* or paediatric* or juvenile or toddler* or kids or girl* or boy* or school age* or minors or adolescent* or teen* or youth).ti,ab,kf. |
| 24 | 22 or 23 |
| 25 | 5 and 9 and 12 and 15 and 21 and 24 |
| 26 | transition to adult care/ or (transition to adult care or transition from child to adult health service* or transition from pediatric to adult care).ti,ab,kf. |
| 27 | ((adult healthcare or adult health care or adult care or adult service* or young adult* or adulthood) not parent*).ti,ab,kf. |
| 28 | 26 or 27 |
| 29 | 25 not 28 |
| 30 | limit 29 to conference abstract status |
| 31 | 29 not 30 |

PSYCINFO (OVID)

Database(s): APA PsycInfo 
Search Strategy:

| # | Searches |
| --- | --- |
| 1 | health care services/ or hospital discharge/ or discharge planning/ |
| 2 | (transition* or discharge or co-ordinated service* or coordinated service* or pediatric care coordinat* or paediatric care coordinat*).ti,ab,id. |
| 3 | (transfer* adj2 (care* or patient)).ti,ab,id. |
| 4 | 1 or 2 or 3 |
| 5 | home care/ or hospice/ |
| 6 | (medical home* or home care or unit* or clinic or service* or program* or framework* or model or hospice).ti,ab,id. or home.ti. |
| 7 | ((hospital or transition* or discharge or nicu or picu) adj3 home).ti,ab,id. |
| 8 | 5 or 6 or 7 |
| 9 | psychological needs/ or needs/ or need satisfaction/ or needs assessment/ or exp experience level/ or knowledge level/ or self-knowledge/ or exp emotional states/ or exp health personnel attitudes/ |
| 10 | (need* or challeng* or prefer* or wish* or obstacle* or worry or worries or confidence or experienc* or satisf* or empower* or self-management or education or perception* or view*).ti,ab,id. |
| 11 | 9 or 10 |
| 12 | exp health personnel/ or caregivers/ |
| 13 | (healthcare provider* or health care provider* or health personnel or medical personnel or medical staff or nurs* or doctor* or physician* or medical specialist* or clinician* or hospital-based clinician* or healthcare worker* or health care worker* or healthcare professional* or health care professional* or pediatrician* or paediatrician* or health administrator* or hospital personnel or hospital staff or health caregiver* or healthcare giver* or social worker* or psychosocial worker* or psychologist* or allied healthcare professional* or speech therapist* or rehabilitation physician* or physical therapist* or home nurse* or caregiver*).ti,ab,id. |
| 14 | 12 or 13 |
| 15 | exp multiple disabilities/ or disabilities/ or disability management/ or neonatal intensive care/ or (chronic* or medical complex* or medically complex* or comorbid* or co-morbid* or multiple morbidit* or complex medical need* or complex medical condition* or special medical condition* or complex health condition* or complex need* or complex care* or complex care need* or complex health need* or complex healthcare need* or complex health care need* or CSHCN or CSHCNs or CMC or yshcn* or disabled child* or medical fragil* or resource intensive need* or extensive health care need* or technology dependen* or complex technological health care need* or complex technological healthcare need* or special health care need* or complex multidisciplinary health*).ti,ab,id. |
| 16 | pediatrics/ or child care/ or chronically ill children/ |
| 17 | (child* or infan* or neonat* or newborn* or baby or babies or preschool or preteen* or schoolchild* or pediatric* or paediatric* or juvenile or toddler* or kids or girl* or boy* or school age* or minors or adolescent* or teen* or youth).ti,ab,id. |
| 18 | 16 or 17 |
| 19 | 4 and 8 and 11 and 14 and 15 and 18 |
| 20 | (adult care or adult* or midwifery or child abuse).ti,ab,id. or child abuse/ or public health/ or exp military personnel/ or exp "racial and ethnic groups"/ or exp criminal behavior/ |
| 21 | (transition to adult care or transition from child to adult health service* or transition from pediatric to adult care).ti,ab,id. |
| 22 | ((adult healthcare or adult health care or adult care or adult service* or young adult* or adulthood) not parent*).ti,ab,id. |
| 23 | 20 or 21 or 22 |
| 24 | 19 not 23 |

CINAHL (Ebsco):

( (MH "Hospital to Home Transition")  OR (MH "Transitional Care") OR ( MH "Discharge Planning+") OR (MH "Child Health Services+/AM/OG") OR TI ( transition* or discharge or co-ordinated service* or coordinated service* or pediatric care coordinat* or paediatric care coordinat*) OR AB ( transition* or discharge or co-ordinated service* or coordinated service* or pediatric care coordinat* or paediatric care coordinat*)

AND

( (MH "Home Health Care") OR (MH "Hospice Care") OR TI home* ) OR ( TI ( medical home* or home care or unit* or clinic or service* or program* or framework* or model or hospice ) OR AB ( medical home* or home care or unit* or clinic or service* or program* or framework* or model or hospice ) ) OR ( TI ( (hospital or transition* or discharge or nicu or picu) N3 home ) OR AB ( (hospital or transition* or discharge or nicu or picu) N3 home ) )

AND

(MH "Health Personnel+") OR (MH "Caregivers") OR TI (healthcare provider* OR health care provider* OR health personnel OR medical personnel or medical staff OR nurs* OR doctor* OR physician* OR medical specialist* OR clinician* OR hospital-based clinician* OR healthcare worker* OR health care worker* OR healthcare professional* OR health care professional* OR pediatrician* OR paediatrician* OR health administrator* OR hospital personnel OR hospital staff OR health caregiver* OR healthcare giver* OR social worker* OR psychosocial worker* OR psychologist* OR allied healthcare professional* OR speech therapist* OR rehabilitation physician* OR physical therapist* OR home nurse* OR caregiver*) OR AB (healthcare provider* OR health care provider* OR health personnel OR medical personnel or medical staff OR nurs* OR doctor* OR physician* OR medical specialist* OR clinician* OR hospital-based clinician* OR healthcare worker* OR health care worker* OR healthcare professional* OR health care professional* OR pediatrician* OR paediatrician* OR health administrator* OR hospital personnel OR hospital staff OR health caregiver* OR healthcare giver* OR social worker* OR psychosocial worker* OR psychologist* OR allied healthcare professional* OR speech therapist* OR rehabilitation physician* OR physical therapist* OR home nurse* OR caregiver*)

AND

( (MH "Needs Assessment") OR (MH "Perception+") OR (MH "Emotions+") OR (MM "Health Services Needs and Demand")  OR (MH "Attitude of Health Personnel+") OR ( TI (need* OR challeng* OR prefer* OR wish* OR obstacle* OR worry OR worries OR confidence OR experienc* OR satisf* OR empower* OR self-management OR education OR perception* OR view*) OR AB (need* OR challeng* OR prefer* OR wish* OR obstacle* OR worry OR worries OR confidence OR experienc* OR satisf* OR empower* OR self-management OR education OR perception* OR view*)) )

AND

( (MH "Child, Disabled") OR (MH "Child, Medically Fragile")  OR ( TI (chronic* or medical complex* or medically complex* or comorbid* or co-morbid* or multiple morbidit* or complex medical need* or complex medical condition* or special medical condition* or complex health condition* or complex need* or complex care* or complex care need* or complex health need* or complex healthcare need* or complex health care need* or CSHCN or CSHCNs or CMC or yshcn* or disabled child* or medical fragil* or resource intensive need* or extensive health care need* or technology dependen* or complex technological health care need* or complex technological healthcare need* or special health care need* or complex multidisciplinary health*) OR AB ( chronic* or medical complex* or medically complex* or comorbid* or co-morbid* or multiple morbidit* or complex medical need* or complex medical condition* or special medical condition* or complex health condition* or complex need* or complex care* or complex care need* or complex health need* or complex healthcare need* or complex health care need* or CSHCN or CSHCNs or CMC or yshcn* or disabled child* or medical fragil* or resource intensive need* or extensive health care need* or technology dependen* or complex technological health care need* or complex technological healthcare need* or special health care need* or complex multidisciplinary health* ) ) ) OR ( TI ( (medical* or need* or health condition* or care or disease*) N3 complex* ) OR AB ( (medical* or need* or health condition* or care or disease*) N3 complex* ) ) OR ( TI ( (special or extensive) N3 need* ) OR AB ( (special or extensive) N3 need* ) ) OR TI (neonatal intensive care unit* and transit*)

AND

(MH "Pediatrics+") OR (MH "Child+") OR (MH "Infant+") OR (MH "Adolescence+") OR TI ( child* OR infan* OR neonat* OR newborn* OR baby OR babies OR preschool OR preteen* OR schoolchild* OR pediatric* OR paediatric* OR juvenile OR toddler* OR kids OR girl* OR boy* OR school age* OR minors OR adolescent* OR teen* OR youth ) OR AB ( child* OR infan* OR neonat* OR newborn* OR baby OR babies OR preschool OR preteen* OR schoolchild* OR pediatric* OR paediatric* OR juvenile OR toddler* OR kids OR girl* OR boy* OR school age* OR minors OR adolescent* OR teen* OR youth)

NOT

( MH "Aged, 80 and Over" ) OR TI ( midwifery OR adult* OR transition to adult care or transition from child to adult health service* or transition from pediatric to adult care ) OR AB ( midwifery OR adult* OR transition to adult care or transition from child to adult health service* or transition from pediatric to adult care

**Appendix 2: characteristics of included studies**

Table 2 Characteristics of included studies

| **Author** | **Year** | **Country** | **Study objectives** | **Study method** | **Study setting** | **Number of HCP included** | **Type of HCP** | **Data collection for experiences HCP** |
| --- | --- | --- | --- | --- | --- | --- | --- | --- |
| Abbott et al., [2] | 2005 | UK | To examine the impact of multi-agency working on professionals supporting disabled children with complex healthcare needs. | Qualitative study | Multi-agency services (urban and rural) | 115 | - (specialized) nurses - Pediatricians / medical specialists - Allied health care professionals - Case managers / care coordinators - Psychologists - Social workers - Administrative staff, clinical directors and/or managers - Others | Semi-structured interviews |
| Abebe et al., [3] | 2020 | USA | To describe characteristics of clinical care notes and highlight current gaps in the context of a pediatric complex care program. | Qualitative study | Childrens hospital | 35 | - (specialized) nurses - Pediatricians / medical specialists - Case managers / care coordinators | Observations of provider-family interactions and focus group with HCP |
| Adams et al., [4] | 2013 | Canada | To understand the usefulness and desired content of comprehensive care plans by exploring the perceptions of parents and HCPS of CMC. | Qualitative study | Pediatric academic hospital | 15 | - (specialized) nurses - Pediatricians / medical specialists | Focus groups |
| Babayan et al., [5] | 2023 | Canada | To establish the economic, operational, and technical feasibility of piloting the expansion of an existing nurse-led after-hours virtual care service offered to home and community care providers to family caregivers of children with newly inserted medical devices after hospital discharge at Toronto’s Hospital for Sick Children. | Mixed method study | Pediatric academic hospital | 28 | - (specialized) nurses - Administrative staff, clinical directors and/or managers - Others | Semi-structured interviews |
| **Author** | **Year** | **Country** | **Study objectives** | **Study method** | **Study setting** | **Number of HCP included** | **Type of HCP** | **Data collection for experiences HCP** |
| Barnard et al., [6] | 2013 | USA | This study is a qualitative investigation of programme, surgical and at home recovery experiences among CSHCNs and their family carers who participated in a spine surgical care programme at a paediatric hospital in the Western United States. | Qualitative study | Pediatric hospital | 14 | - (specialized) nurses - Pediatricians / medical specialists - Allied health care professionals - Social workers - Administrative staff, clinical directors and/or managers | Semi-structured in depth interviews |
| Brenner et al., [7] | 2018 | EU | This viewpoint presents and discusses the development of the first core principles and standards for effective, personalised care of CCCNs. | Mixed method study | Hospital and community setting | Not specified, HCP of 23 countries participated. | Not reported | Reviews, case studies, surveys, document analysis, interviews, care process analysis |
| Brenner et al., [8] | 2018 | EU | To examine current approaches to care management of CCCNs in 30 EU and EEA countries, and the implications for primary care service delivery. |  |  |  |  | Questionnaire |
| Brenner et al., [9] | 2020 | EU | This study examined the structures and processes of care in place for CCCNs and identified key constituents for effective integration of care for these children at the community and acute care interface across 30 EU/EEA countries. |  |  |  |  | Surveys with closed and open ended questions |
| Carter et al., [10] | 2016 | UK | To explore the work of nurses whose main role is supporting children and families move from an institutional place of care to long-term care within the family home. | Qualitative study | Hospital and community setting | 46 | - (specialized) nurses - Pediatricians / medical specialists - Allied health care professionals - Psychologists - Social workers - Others | Face-to-face interviews |
| **Author** | **Year** | **Country** | **Study objectives** | **Study method** | **Study setting** | **Number of HCP included** | **Type of HCP** | **Data collection for experiences HCP** |
| Coller et al., [11] | 2017 | USA | To conduct a rigorous, structured process to develop intervention strategies aiming to reduce hospitalizations within a complex care program population | Mixed method study | Hospital and community setting | 9 | - (specialized) nurses - Pediatricians / medical specialists | Panel discussion |
| Coller et al., [12] | 2020 | USA | To establish priorities for a national research agenda for CYSHCN through a structured, multistakeholder, mixed-methods approach. | Mixed method study | Hospital and community setting | 219 | - (specialized) nurses - Pediatricians / medical specialists - Allied health care professionals - Psychologists - Administrative staff, clinical directors and/or managers - Others | Open-ended survey and panel discussion |
| Cuevas-Asturias et al., [13] | 2024 | UK | To analyze bed occupancy, examine current practice, and explore ideas to improve PICU care of patients with long term and additional needs. | Mixed method study | Academic hospital (PICU) | 16 | - (specialized) nurses - Pediatricians / medical specialists - Others | Survey with closed and open ended questions |
| Curran et al., [14] | 2020 | Canada | To develop recommendations to improve the transition from hospital to home for children with complex and medically fragile needs in Nova Scotia. | Mixed method study | Pediatric tertiaty care facility | 36 | - (specialized) nurses - Pediatricians / medical specialists - Allied health care professionals - Administrative staff, clinical directors and/or managers - Others | Interviews and focusgroups |
| Dallas et al., [15] | 2023 | USA | This program evaluation explores parent and provider experiences of a novel longitudinal care coordination program for infants with medical complexity from the NICU through their first year of life post-discharge | Mixed method study | Hospital and community setting | 34 | - (specialized) nurses - Pediatricians / medical specialists - Allied health care professionals - Case managers / care coordinators - Social workers - Others | Semistructured interviews and focusgroups |
| **Author** | **Year** | **Country** | **Study objectives** | **Study method** | **Study setting** | **Number of HCP included** | **Type of HCP** | **Data collection for experiences HCP** |
| Fratantoni et al., [16] | 2019 | USA | Our goal for this article is to strengthen pediatrician understanding of the PHHC process, highlighting key points in which pediatricians can facilitate services for patients. | Qualitative study | Hospital and community setting | 45 | - (specialized) nurses - Pediatricians / medical specialists - Allied health care professionals - Case managers / care coordinators - Social workers - Others | Semi-structured interviews |
| Glick et al., [17] | 2024 | USA and Canada | To examine pediatrician perspectives on barriers and facilitators experienced by parents in comprehension of and adherence to inpatient discharge instructions for CMC | Qualitative study | Hospital | 20 | - Pediatricians / medical specialists | Semi-structured interviews |
| Gorsky et al., [18] | 2023 | USA | To describe the role of uncertainty (1) during the NICU experience and during the transition-to-home period, (2) accessing community-based services, and (3) addressing mental health. | Qualitative study | Academic hospital (NICU) | 29 | - (specialized) nurses - Pediatricians / medical specialists - Allied health care professionals - Case managers / care coordinators - Psychologists - Social workers - Others | Semi-structured interviews |
| Gupta et al., [19] | 2004 | USA | To examine the frequency with which pediatricians provide care coordination services to children, particularly those with special health care needs, in their practices and the barriers to providing these services | Quantiative study | Members of AAP | 803 | - Pediatricians / medical specialists | Closed ended questionnaire |
| Henderson et al., [20] | 2017 | USA | We interviewed participants who routinely care for children with chronic critical illness to describe their experiences with ICU care for pediatric chronic critical illness. | Qualitative study | Hospital and community setting | 44 | - Pediatricians / medical specialists - Administrative staff, clinical directors and/or managers | Semi-structured interviews |
| **Author** | **Year** | **Country** | **Study objectives** | **Study method** | **Study setting** | **Number of HCP included** | **Type of HCP** | **Data collection for experiences HCP** |
| Kirk et al., [21] | 1999 | UK | 1) to explore the experiences of families caring at home for a technology-dependent child, 2) to identify problems and good practice in the purchasing, delivery and coordination of services, and 3) to assess the appropriateness and adequacy of support services, particularly the specialist expertise amongst generic community-based health professionals and the coordination of multiple-service inputs for families with very complex needs. | Qualitative study | Hospital and community setting | 44 | - (specialized) nurses - Pediatricians / medical specialists - Social workers - Others | In-depth interviews |
| Kirk et al., [22] | 2001 | UK | To explore parents' experiences of caring for a technology-dependent child and of the professionals supporting them in the community. |  |  |  |  |  |
| Kirk et al., [23] | 2002 | UK | The study investigated what support parents wanted, what they actually received and how congruent these were with professionals’ perception. |  |  |  |  |  |
| Kirk et al., [24] | 2004 | UK | To explore the experiences of families caring at home for a technology-dependent child; to examine their needs for practical and other support; and to examine how far services are currently meeting these needs. |  |  |  |  |  |
| Kobussen et al., [25] | 2020 | Canada | To examine perspectives of pediatric intensive care unit health care providers regarding pediatric patients with complex chronic conditions, and to explore potential opportunities to improve these patients’ care. | Mixed method study | Academic hospital (PICU) | 10 | - (specialized) nurses - Pediatricians / medical specialists - Rehabilitation physician - Allied health care professionals | Survey and semistructured interviews |
| **Author** | **Year** | **Country** | **Study objectives** | **Study method** | **Study setting** | **Number of HCP included** | **Type of HCP** | **Data collection for experiences HCP** |
| Law et al., [26] | 2011 | Scotland | This paper is a report of a descriptive qualitative study of the role and activities of nursing and allied health professionals caring for children with complex needs in a community setting. | Qualitative study | Home care | 26 | - (specialized) nurses - Allied health care professionals | Semi-structured interviews and focusgroups |
| Leyenaar et al., [27] | 2017 | USA | To examine the scope of preferences, priorities, and goals of parents of CMC regarding planning for hospital-to-home transitions and to ascertain health care providers’ perceptions of families’ transitional care needs. | Qualitative study | Hospital- and ambulatory-based health care providers | 16 | - (specialized) nurses - Pediatricians / medical specialists | Semi-structured interviews |
| Leyenaar et al., [28] | 2018 | USA | To identify important and feasible hospital to home transitional care interventions for CMC from the perspectives of parents and health care professionals | Mixed method study | Inpatient, hospital-based outpatient and community-based | 37 | - (specialized) nurses - Pediatricians / medical specialists - Case managers / care coordinators - Social workers | Delphi with closed and open ended questions |
| Manhas et al., [29] | 2012 | Canada | To examine how the traditional norms of hospital and home morph into the unique responsibilities of complex pediatric home care | Qualitative study | Hospital and community | 23 | - (specialized) nurses - Pediatricians / medical specialists - Case managers / care coordinators - Social workers - Administrative staff, clinical directors and/or managers | Semi-structured interviews |
| Manhas et al., [30] | 2015 | Canada | To conduct an ethical inquiry into the role and responsibilities of nurses in the qualitative experience of adults involved in the hospital-to-home transition of young, ventilator-dependent children. |  |  |  |  |  |
| **Author** | **Year** | **Country** | **Study objectives** | **Study method** | **Study setting** | **Number of HCP included** | **Type of HCP** | **Data collection for experiences HCP** |
| McLorie et al., [31] | 2023 | UK | To identify models of care for CMC and barriers and facilitators to delivering high-quality care for this population from a ‘free at point of care’ national health service | Qualitative study | Tertiary and non-tertiary hospitals with PICU | 37 | - (specialized) nurses - Pediatricians / medical specialists - Case managers / care coordinators - Administrative staff, clinical directors and/or managers | Semi-structured interviews |
| Nageswaran et al., [32] | 2020 | USA | To describe issues related to transitioning CMC from hospital to home health care and identify strategies to improve this transition. | Qualitative study | Tertiary care children's hospital and home care | 32 | - (specialized) nurses - Pediatricians / medical specialists - Case managers / care coordinators - Social workers - Administrative staff, clinical directors and managers - Others | Focus groups |
| Nelson et al., [33] | 2023 | USA | To explore the perspectives of pediatric hospitalists and pediatric primary care providers within our health system. | Quantiative study | Hospital and community | 78 | - (specialized) nurses - Pediatricians / medical specialists - Case managers / care coordinators - Social workers | Survey with closed ended questions |
| Noyes et al., [34] | 2014 | Ireland | To report a novel review to develop a health systems model of successful transition of children with complex healthcare needs from hospital to home. | Mixed method study | n/a | not reported | not reported | Group discussions |
| **Author** | **Year** | **Country** | **Study objectives** | **Study method** | **Study setting** | **Number of HCP included** | **Type of HCP** | **Data collection for experiences HCP** |
| Price et al., [35] | 2018 | UK | What are parent and professional perspectives of hospice step-down care within a constituent country of the UK? | Qualitative study | Hospital and community setting | 26 | - (specialized) nurses - Pediatricians / medical specialists - Allied health care professionals - Social workers - Administrative staff, clinical directors and managers | Focus groups |
| Ramalho et al., [36] | 2022 | Brasil | To analyze the nurse’s performance in the hospital discharge process of children with chronic disease. | Qualitative study | Pediatric unit of a public hospital | 10 | - (specialized) nurses | Semi structured interviews |
| Ravid et al., [37] | 2020 | USA | Evaluation of introduction of a multidisciplinary videoconference with patients, parents, HCP in hospital and in home situation shortly before hospital discharge | Qualitative study | Hospital | 5 | - (specialized) nurses - Pediatricians / medical specialists | Field notes, conducted surveys and semi-structured interviews. |
| Sobotka et al., [38] | 2020 | USA | Hospital discharge practices and home health services are not standardized for children with invasive home mechanical ventilation (HMV). We assessed discharge practices for U.S. children with HMV. | Mixed method study | Hospital | 59 | - (specialized) nurses - Pediatricians / medical specialists - Case managers / care coordinators | Questionnaires with closed and open ended questions |
| Tearl et al., [39] | 2006 | USA | Evaluation of parent and HCP satisfaction of single discharge coordinator and clinical pathway compared to different HCP's. | Mixed method study | Hospital | 17 | - (specialized) nurses - Pediatricians / medical specialists - Social workers | Survey and interview |
| Tearl et al., [40] | 2007 | USA | Evaluation of satisfaction of parents and companies with single dedicated respiratory therapist coordinator | Quantitative study | Hospital | Not reported | - Administrative staff, clinical directors and managers - Others | Survey |
| Van de Riet et al., [41] | 2024 | Netherlands | To gain deeper insights into the H2H transition process and to work towards eHealth interventions for its improvement, by applying an iterative methodology involving both CMC families and HCP as end-users | Qualitative study | Pediatric academic hospital | Not reported | - (specialized) nurses - Pediatricians / medical specialists - Rehabilitation physician | Participant observations, semi structured interviews and focus groups |

**Appendix 3: Overview of identified themes, categories and a selection of open coding illustrated by citations**

| **Themes (n = 11)** | **Categories (n= 24)** | **Open coding (n= 434)*** | **Citations** |
| --- | --- | --- | --- |
| **1. Competencies and knowledge about H2H care** | Knowledge about providing H2H care | Specialized field requiring specific expertise  No insight how life looks like for families at home  Unaware of existing initiatives (support in community/formal guidelines) | “The majority of participants were not aware of formal guidelines or policies to help support or inform the transition from hospital to home for children with medical complexity and their families.” (Curran 2020)  “You know, I guess the big thing in our community is knowing what’s out there for them, even from an acute care side, whether it’s the [pediatric tertiary care facility]. If you have a child that’s going home, being discharged from the [pediatric tertiary care facility] you don’t necessarily know all the supports that are in all the home communities that these children are going to. And I think we’re the same way, is that we don’t always realize all the supports that are in place.” (Curran 2020) |
|  | Training and education for HCP | Need for training pathway | "[ICU staff] discharge kids without adequate support. They don’t have a vision of what their lives are like. They need the training. I’ve had difficulty getting critical care fellows and residents to come see what happens with these families once they go home. They need the insight; they need to be familiar with really what happens." (Henderson, 2017) |
| **2. Emotional burden for HCP** | Concerns about families transitioning home | Complex process for families  From 24/7 care to home  Siblings | “The most difficult thing for them is that they suddenly have gone from a very, very controlled environment where there’s lots of monitors and lots of people watching their  baby, to them being all alone and feeling resource poor and vulnerable.” (Leyenaar 2017) |
|  | Lack of confidence | Limited experience with care | "How do you judge where a family’s comfort level is with discharge and training? Because you can feel like you’ve checked off all the boxes on the checklist of training, but the family gets home and they feel uncomfortable and within 48 hours they’re readmitted." (Henderson, 2017). |
|  | Time pressure | Pressure to free a bed  Time constraints  Overwhelming workloads | We’re under increasing pressure from the hospital side about blocking beds, needing to move, winter’s coming in, infections are rife, and it’s move, move, move. So, we’re being propelled and driven along a road at a rate that...the two things are at odds necessarily with each other (Price 2018)  Participants acknowledged that the inpatient team is often inaccessible, limiting time allocated for comprehensive discharge counseling and for families to ask questions. (Glick 2024) |
| **3. Parent - HCP relationship** | Bond between HCP and parents | Continuity  Strong connection | Continuity in parent-professional relationships was identified by both parents and professionals as being important in developing a relationship characterised (Kirk 2002)  The nurses talked about how they “pulled things together” for families by dedicating time to build in-depth relationships with the families and the stakeholders across a variety of settings. (Carter 2016 - Knowing the places of care, p. 10) |
|  | Acknowledgement emotional needs parents | Acknowledgment of emotional needs  Blurred boundaries | “I think that normalization of the things that parents experience, like the significant rollercoaster and the constant uncertainty I think that people feel, like, “What will my baby be like? What will it be like at home?”, “Will my baby be able to go to school normally,” and those kinds of uncertainties. . . . But having something to address that might be helpful as an adjunctive to things like family meetings and social support from the social worker in the unit or something”. |
| **4. Communication with parents** | Tailoring communication to parental level | Equality  Language barriers  Learning difficulties  Culture sensitive | Linguistically appropriate information was identified as a key constituent to effective individualised care, to sup-port equity of care and optimum readiness for caring for the child at home. (Brenner 2020)  "People learn information in multiple different ways, and so, your strategy should be to present the information in multiple different ways. So, I always have a written or visual component, ideally with pictures…in the primary language of the patient, and then I also verbally go over it… It can't just be spoken word" (Glick, 2024). |
|  | Clear expectations | Hard road home  Realistic  Expectations regarding parental responsibilities | "Providers described the importance of 'really helping parents understand what to expect and setting out a realistic course.' With respect to ongoing child development, parents prioritized their child’s growth, weight gain, and emotional and physical development" (Leyenaar, 2017). |
| **5. Communication among HCP** | Responsibilities | Clear about who is responsible  Shifting across transition  Need for care coordinator | Participants reported that parents may not know which specialist is responsible for which issues (e.g., refilling specific prescriptions) (Glick 2024)  "The transition period was considered a difficult period because of the lack of clarity about the providers in charge of the child’s care. Home health care providers often did not know whom to contact if there was a problem after discharge. Some mentioned having the contact number for the attending physician to call if they had a question" (Nageswaran, 2020).  Even professionals themselves were sometimes unclear about their responsibilities; both GPs and nurses were unsure whether GPs or hospital consultants were medically responsible for children while at home. (Kirk 2003)  Accountability and liability for aspects of care of these children and families shifted along transition (Manhas 2012)  Participants did report, however, that it could be difficult when NICU inpatient providers, especially trainees or mid-level providers, and care coordinators were both trying to coordinate procedures or outpatient appointments. If another provider started scheduling follow-up, the care coordinator’s recommendation could duplicate work or cause tension among staff. (Dallas 2023) |
|  | Information sharing | Difficult between care settings  No access  Up to date | The findings also reveal the complex work of CCP staff in gathering patient information, updating their care notes, and communicating with clinicians within and outside of the CCP. These tasks are distributed across care settings and time, some of which involve long latency periods (Abebe 2020)  Health care providers also discussed the inability to access certain discharge summaries and patient charts and reported not receiving care plan information in a timely manner (Curran 2020) |
| **6. Holistic care plan** | Standardized care plan | Standard is needed  Continuous and adaptive  Timely | A standardised system is in place to identify the clinical support needs for a child transitioning to home (Brenner 2018)  Professionals noted that both appropriate individualized and specialized short-term care and  home-support services need to be planned in advance, not on a crisis basis, because of the lengthy process involved in recruiting and training carers. (Kirk 1999) |
|  | Identify family needs | Shared decision making  Listen to parents  Work together with parents | I’m in the business of making complex diagnoses that require procedures, surgery, interventions. Then we’re taking the child and handing them to a family who has to support the child at home. So guess what? Their opinion matters. And their resources matter. Things that don’t seem as pertinent to us when we’re making the initial decisions end up being what really determines the baby’s life. (Henderson 2017) |
|  | Social factors of health | Psychosocial challenges in families  Financial challenges  Home environment  Daily routines families | "…we find out that a caregiver has a mental health breakdown or there's a divorce or all of those psychosocial stressors…can end up impacting the care of their children." (Glick 2024)  “Family education and discussions regarding activities of daily living and challenges are critical for a successful transition into the home environment (Kobussen 2020)” |
|  | Care after discharge | Follow up appointments  Phone calls  Home visits  Use of telemedicine | “Follow up with these chronic families. Make a call the next day. How are things going, how was your fi rst night? Just to make them feel like you know . . . we’re still connected. I’m not doing this alone. I think that makes all the difference in the world. Making a follow-up just to make sure. Is there anything we should have done differently? What can we change to make tonight a better night? How can we help you more so you don’t wind up back in the hospital?” (Leyenaar 2017)  Follow-up by telephone every 1-2 days during first week after discharge to address needs, questions and concerns of  caregivers. (Coller 2017) |
| **7. Educational plan** | Standardized manner | Marking / celebrating milestones  Emergencies  Pain and symptom management | …[Hospital staff] don’t ever prepare the family appropriately to handle a true emergency situation...Some of them are over-taught and some of them are briefed and then sent out the door. I think if the process was the same for all families, it would be great. (FG3, N11) (Nageswaran 2020)  Make sure they have a good pain management protocol and an expectation on how the pain should improve after the surgery or admission.” (Leyenaar 2017) |
|  | At parents pace | Gaining parental confidence  Limited by parent availability and time  Hospital not ideal setting | ‘‘[I’ll say] ‘Have a go; if you can’t do it I’ll take overyI’ll phone you at so and so, and see how you’ve got on. And if you can’t do it don’t worry, I’ll just come and help you.’ And then it’s just sort of given them that extra support they’ve got to believe in themselves and it’s just a case of building up their confidence to believe in themselves, that they can do it.’’ (Kirk 2002)  Parents are supported to be clinically ready to care for their child at home, in an incremental manner (Brenner 2018)  The promotion of “family togetherness” was noted as being lar-gely hampered within the hospital with, for example, limited facilities for parents, and when visits from siblings were not permitted. (Price 2018) |
| **8. Facilitating access to care** | Access to home care | Lack of human resources  Hard to keep good trained home HCPs  Differences between municipalities | The whole discharge process, the biggest delay always... is finding trained caregivers. ...I know they did a big blitz where they would hire people just to train them to be pediatric caregivers and pay them while they were going to school. Even after that exercise, [it] didn’t guarantee that they were keeping any of them. ... Some of them might work for a little while and then quit and then you’re back to square one ... Once these families get home ... if they have a caregiver quit, it could be crippling to them where the child has no option, but to go back to hospital because a parent can only do so much. ( Manhas 2015)  Participants agreed that quality pediatric home  health care depends on providers with strong pediatric skills; these providers are in short supply, which can delay hospital discharge or initiation of pediatric home health care services. (Frantantoni 2019) |
|  | Access to resources and equipment | Hard to get equipment home  Differences between hospital and home | …we're sending medications out into the void, and sometimes don't know if the parents are actually going to get these medications. …making sure that all the supplies have been delivered to the house, that all the medications are available at the pharmacy that they don't need prior authorization…that the pharmacy has the medicine in the right form, so, liquid versus a tablet, for example. (Glick 2024) |
|  | Social support | Respite care | Finding, securing, training, and maintaining appropriate paid caregivers to provide respite at home was a universally espoused challenge. (Manhas 2012)  Primary care practices have no adequate social work and care coordination resources necessary to bridge hospital care (Nelson 2023) |
| **9. Housing problems** | Accessible and appropriate housing | No houses available  Lengthy procedure to get appropriate housing | “Very frequently there are electrical issues in the home that need to be fixed before the equipment is in the home” and “A lot of the time, parents need to move because of space in the home and amount of family members currently residing to safely accommodate child and equipment.” (Sobotka 2020)  These are families that are complex in the nature of potentially being homeless, not having appropriate accommodation. It’s housing that takes ages because if there’s no houses, there’s no houses. There’s nowhere to discharge this child. (McLorie 2023) |
| **10. Funding & bureaucratic challenges** | Funding | Obtaining funding  Responsible for funding | “unclear where funding responsibilities for short-term care lie, leading to inconsistent interpretations at the local level and variations in the services available to families (Kirk, 1999).” |
|  | Bureaucratic challenges | Delays in approval | “Despite the best efforts of healthcare professionals, some discharges take months to complete due to delays in arranging care packages, leaving children "stuck" in hospitals. These extended hospital stays often result in negative financial, social, and emotional consequences for families and cause frustration among professionals working within a system they feel powerless to influence (Carter, 2016).” |
| **11. Coordination and continuity** | Coordination and continuity | Value of care coordination | “Many providers also commented on the value of care coordination and check-ins to support families in the transition from the NICU to home. (Gorsky 2023).”  “Gaps in care coordination can interrupt pediatric home health care services. When prescribers lack care-coordination support or experience, families become case managers by default, responsible for identifying home health needs and investigating options. (Fratantoni 2019).” |
| * selection of open codes is presented | | | |

**Appendix 4: Distribution of the eleven themes across the 40 included papers**

| **Overarching themes** | **1. Competencies and knowledge about H2H care** | **2. Emotional burden for HCP** | **3. Parent-HCP relationship** | **4. Communication with parents** | **5. Communication among HCP** | **6. Holistic care plan** | **7. Educational plan** | **8. Facilitating access to care** | **9. Housing problems** | **10. Funding & bureacratic challenges** | **11. Coordination and continuity** |
| --- | --- | --- | --- | --- | --- | --- | --- | --- | --- | --- | --- |
| **Included studies** |  |  |  |  |  |  |  |  |  |  |  |
| Abbott et al., |  |  |  |  |  |  |  |  |  |  |  |
| Abebe et al., |  |  |  |  |  |  |  |  |  |  |  |
| Adams et al., |  |  |  |  |  |  |  |  |  |  |  |
| Babayan et al., |  |  |  |  |  |  |  |  |  |  |  |
| Barnard et al., |  |  |  |  |  |  |  |  |  |  |  |
| Brenner et al., |  |  |  |  |  |  |  |  |  |  |  |
| Brenner et al., |  |  |  |  |  |  |  |  |  |  |  |
| Brenner et al., |  |  |  |  |  |  |  |  |  |  |  |
| Carter et al., |  |  |  |  |  |  |  |  |  |  |  |
| Coller et al., |  |  |  |  |  |  |  |  |  |  |  |
| Coller et al., |  |  |  |  |  |  |  |  |  |  |  |
| Cuevas-Asturias et al., |  |  |  |  |  |  |  |  |  |  |  |
| Curran et al., |  |  |  |  |  |  |  |  |  |  |  |
| Dallas et al., |  |  |  |  |  |  |  |  |  |  |  |
| Fratantoni et al., |  |  |  |  |  |  |  |  |  |  |  |
| Glick et al., |  |  |  |  |  |  |  |  |  |  |  |
| Gorsky et al., |  |  |  |  |  |  |  |  |  |  |  |
| Gupta et al., |  |  |  |  |  |  |  |  |  |  |  |
| Henderson et al., |  |  |  |  |  |  |  |  |  |  |  |
| Kirk et al., |  |  |  |  |  |  |  |  |  |  |  |
| Kirk et al., |  |  |  |  |  |  |  |  |  |  |  |
| Kirk et al., |  |  |  |  |  |  |  |  |  |  |  |
| Kirk et al., |  |  |  |  |  |  |  |  |  |  |  |
| Kobussen et al., |  |  |  |  |  |  |  |  |  |  |  |
| Law et al., |  |  |  |  |  |  |  |  |  |  |  |
| Leyenaar et al., |  |  |  |  |  |  |  |  |  |  |  |
| Leyenaar et al., |  |  |  |  |  |  |  |  |  |  |  |
| Manhas et al., |  |  |  |  |  |  |  |  |  |  |  |
| Manhas et al., |  |  |  |  |  |  |  |  |  |  |  |
| McLorie et al., |  |  |  |  |  |  |  |  |  |  |  |
| Nageswaran et al., |  |  |  |  |  |  |  |  |  |  |  |
| Nelson et al., |  |  |  |  |  |  |  |  |  |  |  |
| Noyes et al., |  |  |  |  |  |  |  |  |  |  |  |
| Price et al., |  |  |  |  |  |  |  |  |  |  |  |
| Ramhalho et al., |  |  |  |  |  |  |  |  |  |  |  |
| Ravid et al., |  |  |  |  |  |  |  |  |  |  |  |
| Sobotka et al., |  |  |  |  |  |  |  |  |  |  |  |
| Tearl et al., |  |  |  |  |  |  |  |  |  |  |  |
| Tearl et al., |  |  |  |  |  |  |  |  |  |  |  |
| Van de Riet et al., |  |  |  |  |  |  |  |  |  |  |  |
| Legend: Black boxes indicate that the corresponding theme was identified in the data extracted from the respective study | | | | | | | | | | | |

**References**

1. Cohen, E., et al., *Children with medical complexity: an emerging population for clinical and research initiatives.* Pediatrics, 2011. **127**(3): p. 529-538.

2. Abbott, D., R. Townsley, and D. Watson, *Multi‐agency working in services for disabled children: what impact does it have on professionals?* Health & social care in the community, 2005. **13**(2): p. 155-163.

3. Abebe, E., et al., *Complexity of Documentation Needs for Children With Medical Complexity: Implications for Hospital Providers.* Hospital pediatrics, 2020. **10**(8): p. 670-678.

4. Adams, S., et al., *Exploring the usefulness of comprehensive care plans for children with medical complexity (CMC): a qualitative study.* BMC Pediatrics, 2013. **13**(1): p. 10.

5. Babayan, K., et al., *An After-Hours Virtual Care Service for Children With Medical Complexity and New Medical Technology: Mixed Methods Feasibility Study.* JMIR pediatrics and parenting, 2023. **6**: p. e41393.

6. Barnard, J.G., et al., *Paediatric spinal fusion surgery and the transition to home-based care: provider expectations and carer experiences.* Health & social care in the community, 2013. **21**(6): p. 634-643.

7. Brenner, M., et al., *Principles for provision of integrated complex care for children across the acute-community interface in Europe.* The Lancet. Child & adolescent health, 2018. **2**(11): p. 832-838.

8. Brenner, M., et al., *Management and integration of care for children living with complex care needs at the acute-community interface in Europe.* The Lancet Child and Adolescent Health, 2018. **2**(11): p. 822-831.

9. Brenner, M., et al., *Key constituents for integration of care for children assisted with long-term home ventilation: a European study.* BMC pediatrics, 2020. **20**(1): p. 71.

10. Carter, B., et al., *“Knowing the Places of Care”: How Nurses Facilitate Transition of Children with Complex Health Care Needs from Hospital to Home.* Comprehensive Child and Adolescent Nursing, 2016. **39**(2): p. 139-153.

11. Coller, R.J., et al., *Strategies to Reduce Hospitalizations of Children With Medical Complexity Through Complex Care: Expert Perspectives.* Academic Pediatrics, 2017. **17**(4): p. 381-388.

12. Coller, R.J., et al., *Health system research priorities for children and youth with special health care needs.* Pediatrics, 2020. **145**(3): p. e20190673.

13. Cuevas-Asturias, S., et al., *A national survey of current practice on patients with long term and additional needs in paediatric intensive care units.* Nursing in critical care, 2024.

14. Curran, J.A., S. Breneol, and J. Vine, *Improving transitions in care for children with complex and medically fragile needs: a mixed methods study.* BMC pediatrics, 2020. **20**: p. 1-14.

15. Dallas, A., et al., *Family and Provider Experiences With Longitudinal Care Coordination for Infants With Medical Complexity.* Advances in neonatal care : official journal of the National Association of Neonatal Nurses, 2023. **23**(1): p. 40-50.

16. Fratantoni, K., et al., *The pediatric home health care process: Perspectives of prescribers, providers, and recipients.* Pediatrics, 2019. **144**(3).

17. Glick, A.F., et al., *Pediatrician perspectives on barriers and facilitators to discharge instruction comprehension and adherence for parents of children with medical complexity.* Journal of Hospital Medicine, 2024. **19**(4): p. 278-286.

18. Gorsky, K.G., et al., *Uncertainty and the NICU Experience: A Qualitative Evaluation of Family and Provider Perspectives.* Children, 2023. **10**(11): p. 1745.

19. Gupta, V.B., K.G. O'Connor, and C. Quezada-Gomez, *Care Coordination Services in Pediatric Practices.* Pediatrics, 2004. **113**(5): p. 1517-1521.

20. Henderson, C.M., et al., *"Stuck in the ICU": Caring for Children With Chronic Critical Illness.* Pediatric Critical Care Medicine, 2017. **18**(11): p. E561-E568.

21. Kirk, S., *Caring for children with specialized health care needs in the community: the challenges for primary care.* Health & social care in the community, 1999. **7**(5): p. 350-357.

22. Kirk, S., *Negotiating lay and professional roles in the care of children with complex health care needs.* Journal of advanced nursing, 2001. **34**(5): p. 593-602.

23. Kirk, S. and C. Glendinning, *Supporting 'expert' parents--professional support and families caring for a child with complex health care needs in the community.* International Journal of Nursing Studies, 2002. **39**(6): p. 625-635.

24. Kirk, S. and C. Glendinning, *Developing services to support parents caring for a technology-dependent child at home.* Child: Care, Health and Development, 2004. **30**(3): p. 209-218.

25. Kobussen, T.A., et al., *Perspectives of Pediatric Providers on Patients With Complex Chronic Conditions: A Mixed-Methods Sequential Explanatory Study.* Critical care nurse, 2020. **40**(5): p. e10-e17.

26. Law, J., D. McCann, and F. O'May, *Managing change in the care of children with complex needs: Healthcare providers' perspectives.* Journal of Advanced Nursing, 2011. **67**(12): p. 2551-2560.

27. Leyenaar, J.A.K., et al., *Families' priorities regarding hospital-to-home transitions for children with medical complexity.* Pediatrics, 2017. **139**(1): p. e20161581.

28. Leyenaar, J.K., et al., *Importance and feasibility of transitional care for children with medical complexity: results of a multistakeholder Delphi process.* Academic pediatrics, 2018. **18**(1): p. 94-101.

29. Manhas, K.P. and I. Mitchell, *Extremes, uncertainty, and responsibility across boundaries: facets and challenges of the experience of transition to complex, pediatric home care.* Journal of child health care : for professionals working with children in the hospital and community, 2012. **16**(3): p. 224-236.

30. Manhas, K.P. and I. Mitchell, *(Dis)-Trust in transitioning ventilator-dependent children from hospital to homecare.* Nursing ethics, 2015. **22**(8): p. 913-927.

31. McLorie, E.V., L. Fraser, and J. Hackett, *Provision of care for children with medical complexity in tertiary hospitals in England: qualitative interviews with health professionals.* BMJ Paediatrics Open, 2023. **7**(1): p. 001932.

32. Nageswaran, S., M.R. Sebesta, and S.L. Golden, *Transitioning children with medical complexity from hospital to home health care: implications for hospital-based clinicians.* Hospital Pediatrics, 2020. **10**(8): p. 657-662.

33. Nelson, A., et al., *Provider Perspectives of Transitions of Care at a Tertiary Care Children's Hospital With a Hospitalist-Run Discharge Clinic.* Clinical Pediatrics, 2023. **62**(8): p. 926-934.

34. Noyes, J., et al., *Reconceptualizing children's complex discharge with health systems theory: novel integrative review with embedded expert consultation and theory development.* Journal of advanced nursing, 2014. **70**(5): p. 975-996.

35. Price, J., S. McCloskey, and K. Brazil, *The role of hospice in the transition from hospital to home for technology‐dependent children—A qualitative study.* Journal of Clinical Nursing, 2018. **27**(1-2): p. 396-406.

36. Ramalho, E.L.R., et al., *Nurse’s performance in the hospital discharge process of children with chronic disease.* Revista Gaúcha de Enfermagem, 2022. **43**: p. e20210182.

37. Ravid, N.L., et al., *Implementation of a multidisciplinary discharge videoconference for children with medical complexity: A pilot study.* Pilot and Feasibility Studies, 2020. **6**(1): p. 27.

38. Sobotka, S.A., et al., *Discharge practices for children with home mechanical ventilation across the United States. Key-informant perspectives.* Annals of the American Thoracic Society, 2020. **17**(11): p. 1424-1430.

39. Tearl, D.K., T.J. Cox, and J.H. Hertzog, *Hospital discharge of respiratory-technology-dependent children: Role of a dedicated respiratory care discharge coordinator.* Respiratory Care, 2006. **51**(7): p. 744-749.

40. Tearl, D.K. and J.H. Hertzog, *Home discharge of technology-dependent children: Evaluation of a respiratory-therapist driven family education program.* Respiratory Care, 2007. **52**(2): p. 171-176.

41. van de Riet, L., et al., *Designing eHealth interventions for children with complex care needs requires continuous stakeholder collaboration and co-creation.* PEC Innovation, 2024. **4**: p. 100280.
